# Osteochondroma-like parosteal osteosarcoma: A case highlighting diagnostic challenge and surgical advances

**DOI:** 10.1016/j.radcr.2024.06.045

**Published:** 2024-07-13

**Authors:** Naveed Majd, Raminta V. Theriault, Morgan A. Darrow, Steven W. Thorpe, Dillon C. Chen

**Affiliations:** a Department of Radiology, University of California Davis Medical Center, 4860 Y Street, Suite 3100, Sacramento, CA 95817, USA; b Department of Orthopedic Surgery, University of California Davis Medical Center, 4860 Y Street, Suite 3800, Sacramento, CA 95817, USA; c Department of Pathology, University of California Davis Medical Center, 4400 V Street, Sacramento, CA 95817, USA; d Department of Orthopedic Surgery, Ochsner Medical Center, 1515 River Road, Jefferson, LA 70121, USA

**Keywords:** Parosteal osteosarcoma, Osteochondroma-like parosteal osteosarcoma, Malignant bone tumor, Sampling error, 3D printing

## Abstract

Parosteal osteosarcomas are uncommon malignant bone tumors that arise from the bone surface. Their heterogenous components can present challenges in diagnosis. We present a case of a rare variant of this tumor known as an osteochondroma-like parosteal osteosarcoma, which was initially misdiagnosed as a cartilaginous tumor on core needle biopsy. Surgical resection of the tumor ultimately allowed for definitive diagnosis. Our case demonstrates the limitations of needle biopsy in diagnosing variants of parosteal osteosarcoma and the vital role of multidisciplinary discussions in guiding diagnosis and treatment. Furthermore, our case utilizes 3-dimensional printing technology in the surgical treatment, and illustrates the recent advances in patient-specific surgical techniques.

## Introduction

Parosteal osteosarcoma (PO) is a rare low-grade malignant bone tumor first described by Geschickter and Copeland in 1957 [1]. It is a surface variant of osteosarcoma arising from the outer periosteum and has an indolent course with a more favorable prognosis compared to conventional osteosarcoma. PO accounts for approximately 5% of osteosarcomas and mainly affects adults in their third and fourth decades of life, with a slightly greater prevalence in females [2,3]. PO usually occurs in the metaphysis, with the most common site being the posterior distal femur, followed by the proximal tibia and proximal humerus [4]. Clinically, it typically presents as a slowly growing, firm painless mass. If it is near a joint, it can cause pain and loss of range of motion [5].

The diagnosis of PO relies heavily on the combination of imaging and pathology.

Radiographically, it appears as an exophytic, lobulated, and centrally ossified mass arising from the cortex. It most commonly occurs at the posterior distal femoral metaphysis. Occasionally a thin lucent line may be seen between the tumor and cortex, known as the string sign [6]. The displacement of normal soft tissue fat planes on radiography suggests an associated soft tissue mass. Computed tomography (CT) and magnetic resonance imaging (MRI) can further evaluate the extent of disease, show intramedullary involvement, assess for associated soft tissue components, and show involvement of important adjacent structures such as a neurovascular bundle [7,8]. CT is superior in evaluating the osseous matrix while MRI best demonstrates marrow and soft tissue involvement. The use of intravenous contrast in imaging can further help identify soft tissue components and marrow involvement [5]. Definitive diagnosis is made with tissue sampling for histologic analysis, which is typically performed under ultrasound or CT guidance. Gross examination of PO typically reveals a broad-based, hard exophytic mass attached to the outer periosteum. There may be a cartilaginous component characterized by scattered cartilaginous foci or an incomplete cartilage cap [2]. Histologic evaluation typically shows linear anastomosing bone trabeculae with intervening paucicellular fibrous tissue containing cytologically atypical spindle cells [9]. Immunohistochemical staining for MDM2 and/or CDK4 may assist with establishing the diagnosis [10]. Sampling error is a known diagnostic pitfall due to the potential heterogeneity within the tumor, which can lead to misdiagnosis if the biopsy is nonrepresentative [11]. The primary treatment of PO is wide surgical resection. Three-dimensional (3D) printing applications have enhanced surgical techniques and allowed for better patient outcomes [12,13].

We present a rare case of an osteochondroma-like PO, which was initially misdiagnosed as a low-grade cartilaginous tumor. Through this case, we aim to provide valuable insights into the complexities of diagnosing this tumor, contribute to the body of knowledge surrounding variants of PO, and bring awareness to the importance of a multidisciplinary approach for optimal outcomes. Furthermore, the use of 3D-printed custom cutting jigs and patient matched allograft in the surgical treatment of our case sheds light on evolving approaches in managing PO.

## Case report

A 24-year-old otherwise healthy and active male presented with intermittent left knee pain for 7 months. Pain was associated with exercise and worsened over the weeks prior to presentation. The patient also endorsed night sweats and weight changes and complaints of a palpable mass in the calf region. Physical exam revealed a firm, palpable mass in the left calf distal to the knee which was tender to palpation. There was associated discomfort with motion. Laboratory evaluation was notable for mildly elevated sedimentation rate (17 millimeters/hour) and bone specific alkaline phosphatase (30.1 micrograms/liter).

**Fig. 1:**
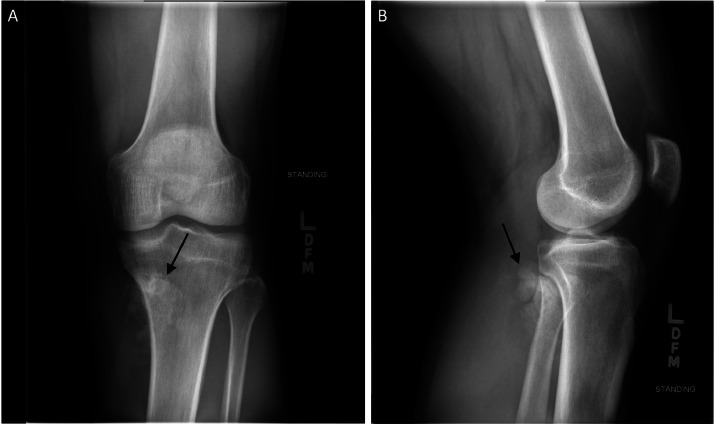
Radiographs of the left knee. (A) Anteroposterior radiograph shows poorly defined osseous mineralization (arrow) about the proximal medial tibia. (B) Lateral radiograph shows an exophytic ossified mass arising from the proximal posterior tibial cortex.

**Fig. 2:**
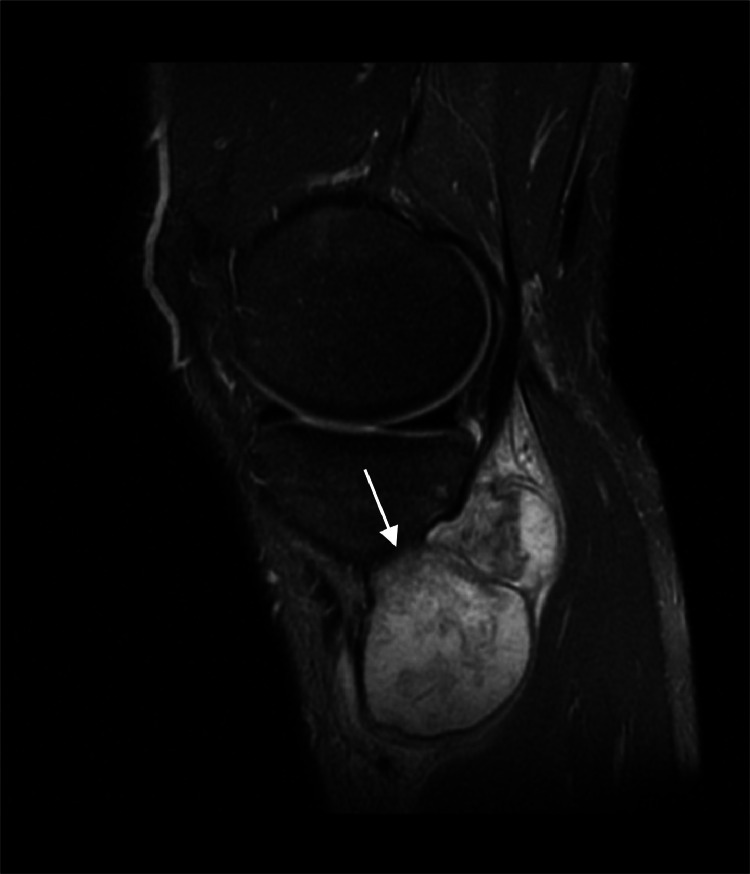
MRI of the left knee. Sagittal T2-weighted image shows a soft tissue mass (arrow) with heterogeneous hyperintense signal contiguous with the cortex of the proximal posteromedial tibial metaphysis.

**Fig. 3:**
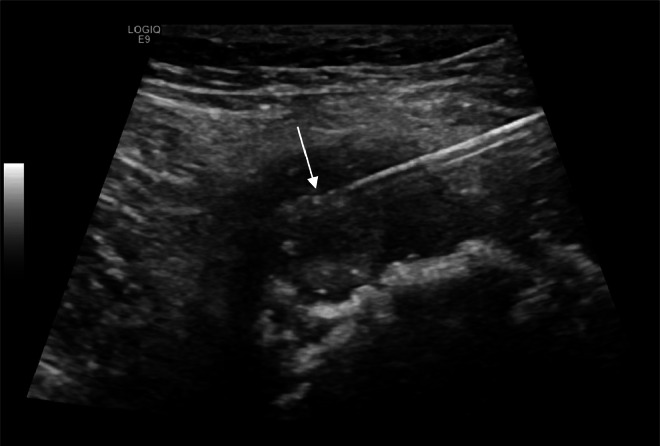
Ultrasound-guided biopsy of the proximal left tibia mass. Grayscale image shows the needle tip (arrow) in the peripheral noncalcified component of the mass. BioPince^TM^ core biopsy device was used with a 16 gauge needle and 23 millimeter throw.

**Fig. 4:**
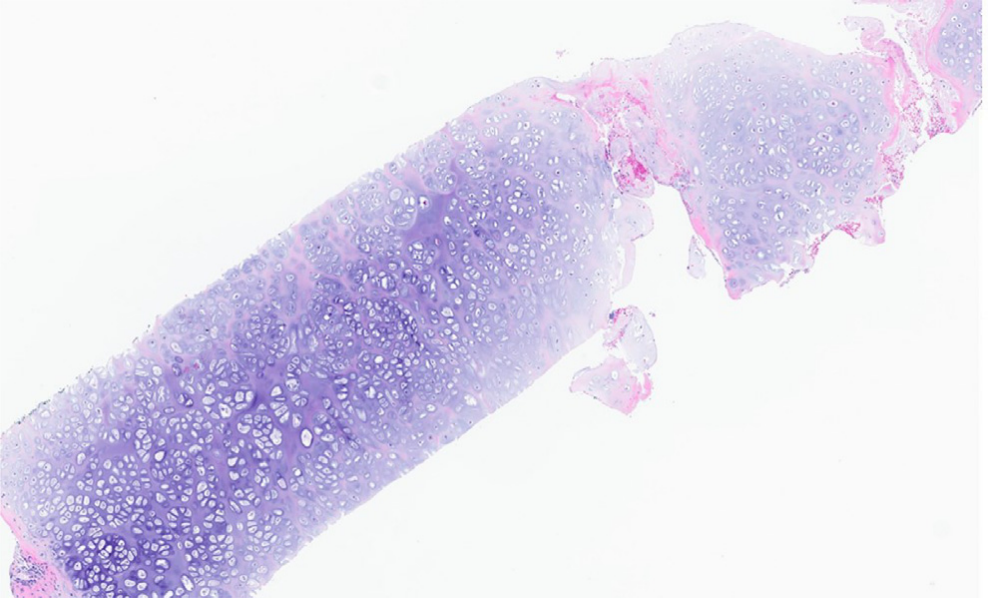
Initial core biopsy showing bland hyaline cartilage (H&E, 100x).

**Fig. 5:**
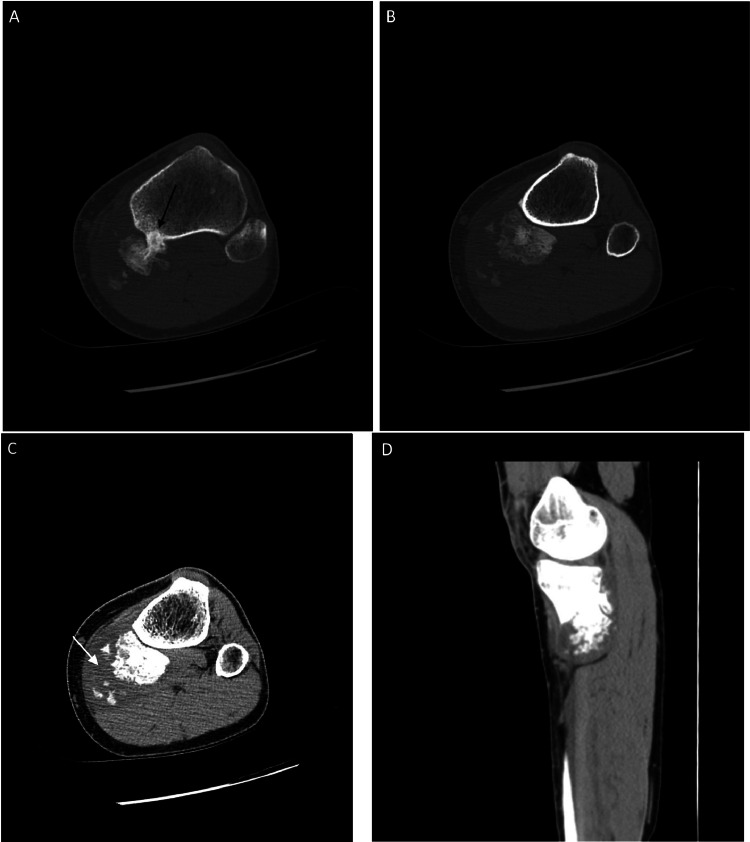
CT of the left tibia. (A and B) Axial bone window images show a parosteal mass at the proximal posteromedial tibia with a small component extending into the medullary space (black arrow) and a central osteoid matrix. (C and D) Axial and sagittal soft tissue window images show the hypodense periphery of the mass (white arrow).

**Fig. 6:**
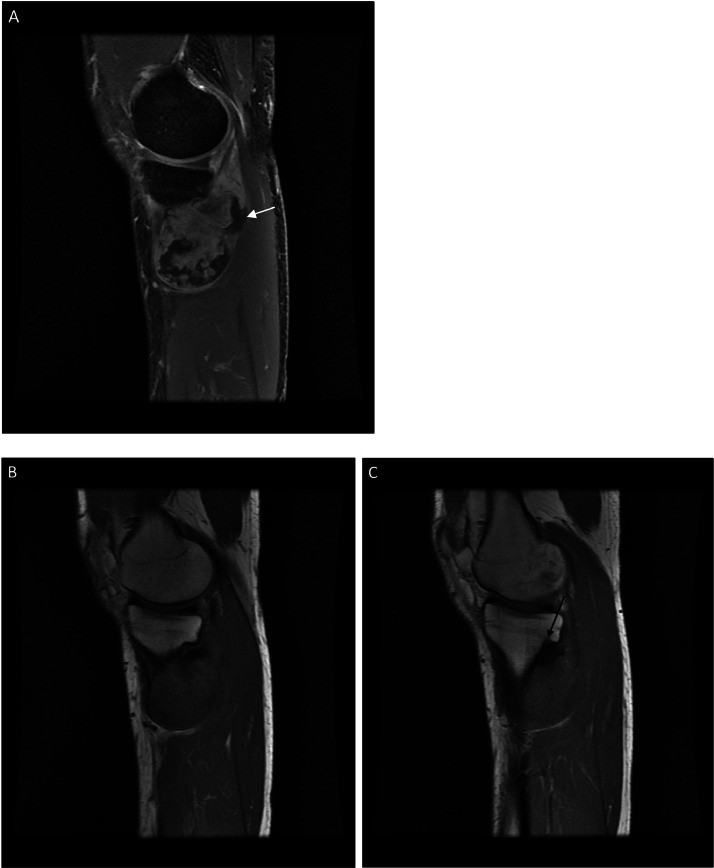
MRI of the left knee. (A) Sagittal fat-saturated T1-weighted image post intravenous contrast shows a heterogeneously enhancing mass arising from the cortex of the posteromedial tibial diaphysis with areas of nonenhancement along its periphery (white arrow). (B and C) Sequential sagittal T1-weighted images demonstrate the low T1 signal of the mass and medullary invasion at its superior portion (black arrow).

Radiographs demonstrated an exophytic mass with an osteoid matrix about the proximal left posteromedial tibia (Figs. 1A and B). MRI without intravenous contrast showed a 5.9 × 5.2 × 4.5 cm soft tissue mass involving the cortex of the proximal posteromedial tibial metaphysis and mild marrow edema in the proximal tibia. The mass was hypointense on T1-weighted images and hyperintense with heterogeneous signal intensity and cystic components on T2-weighted images (Fig. 2). Ultrasound-guided core needle biopsy was subsequently performed using a 16 gauge core biopsy system (BioPince^TM^ device, Argon Medical Devices, Plano, Texas). Sonographic evaluation prior to biopsy showed a heterogeneous mass with scattered internal calcifications. Four samples were obtained from predominantly its noncalcified peripheral component (Fig. 3). Microscopically, the cores consisted of hyaline cartilage with minimal cytologic atypia, foci of reactive woven bone formation, endochondral ossification, and focal necrosis (Fig. 4). Mitoses were inconspicuous. Histologic features of parosteal osteosarcoma, including anastomosing bone trabeculae and atypical cells, were not present. Based on these findings, a diagnosis of low-grade cartilaginous neoplasm was made. The presence of necrosis along with the size of the mass were most suggestive of a periosteal chondrosarcoma, with periosteal chondroma not entirely excluded. CT of the area was obtained for surgical planning and demonstrated slight interval enlargement of the mass over the course of just 5 weeks. The mass was peripherally hypodense with paracentral osteoid mineralization and had a small component extending into the medullary space (Figs. 5A-D). Additional MRI obtained prior to surgical resection, with intravenous contrast (gadobutrol, Bayer HealthCare Pharmaceuticals, Berlin, Germany), showed continued slight enlargement of the heterogeneously enhancing mass and patchy enhancing medullary foci in the proximal tibia (Figs. 6A-C). Of note, CT scan of the chest without contrast was obtained and did not demonstrate any evidence of metastatic disease.

**Fig. 7:**
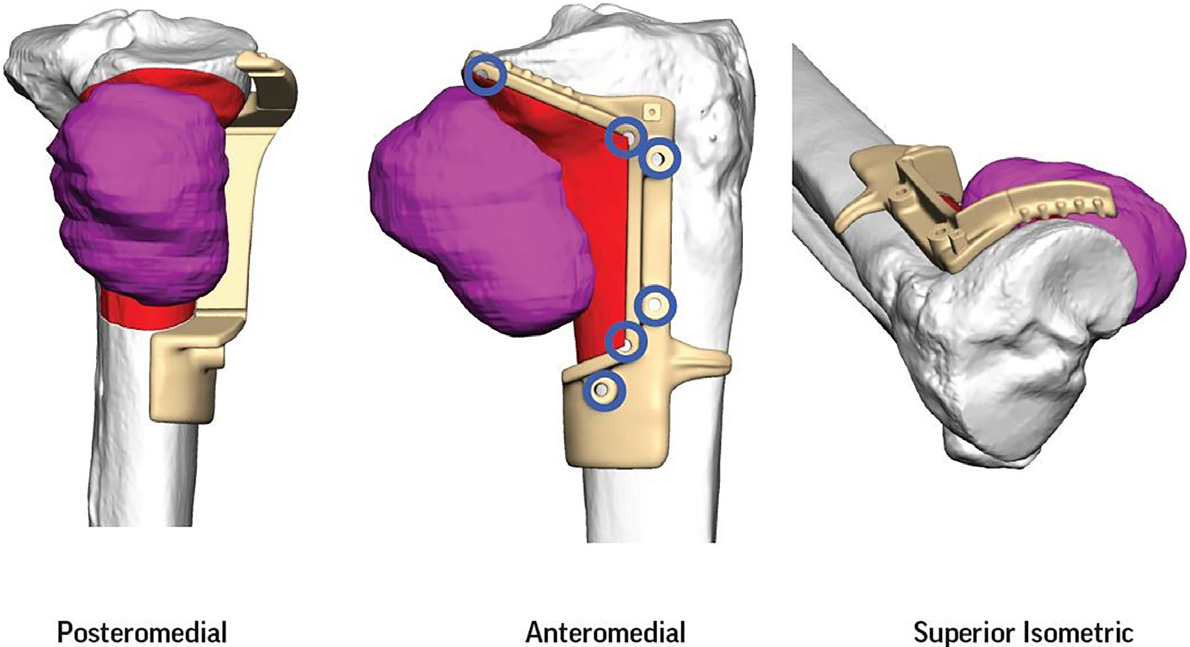
Representative images from digital surgical resection planning with 3D bone modeling and imposed cutting jigs (tan) generated from the patient's imaging, which allowed for precise and adequate resection of the tumor (pink).

**Fig. 8:**
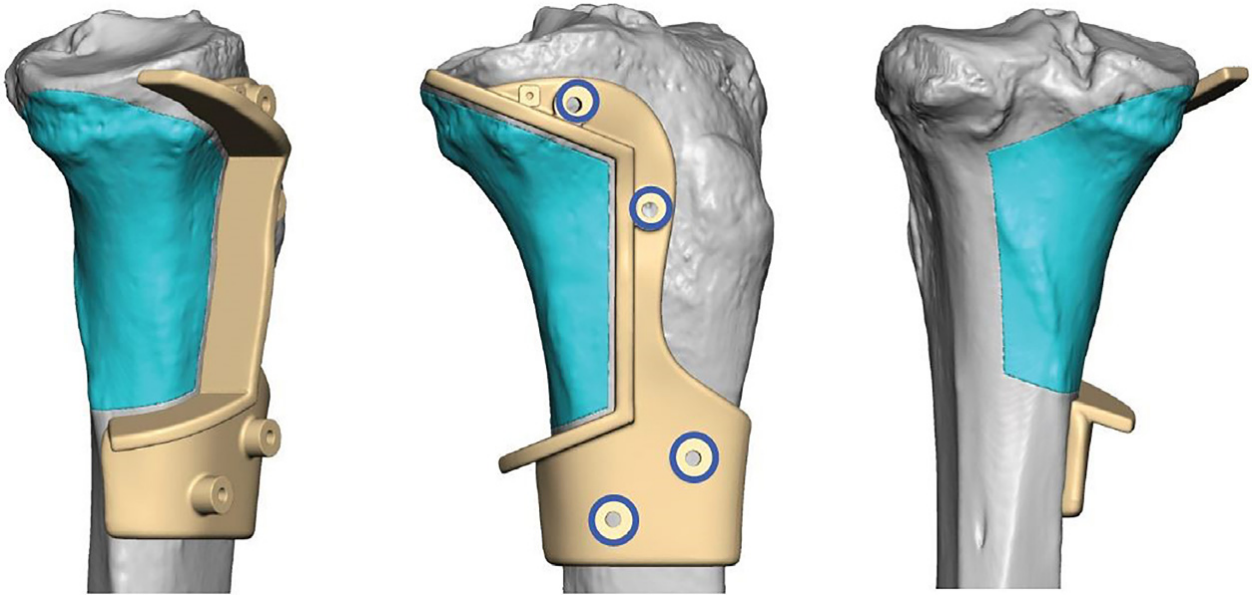
Digital surgical reconstruction planning with 3D bone modeling and cutting jigs, which demonstrates the planned resection from patient and allograft into the tibial defect.

**Fig. 9:**
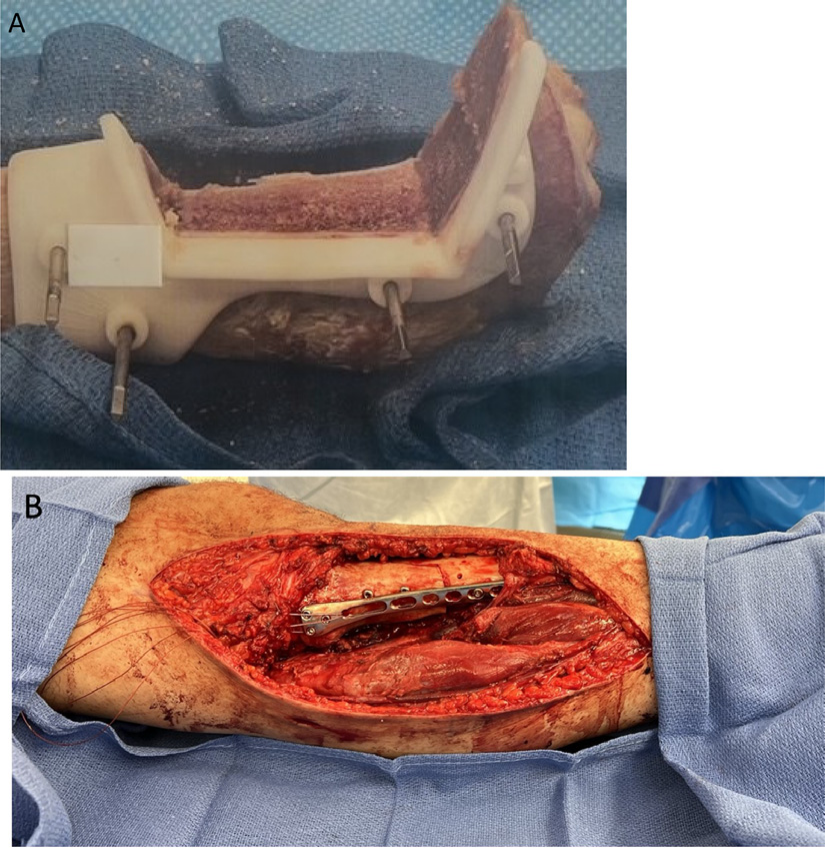
(A) Intraoperative photo of the 3D-printed cutting jig secured on the allograft with segment removed. (B) Intraoperative photo of the reconstructed tibia with the cut allograft segment secured with plate and screws.

**Fig. 10:**
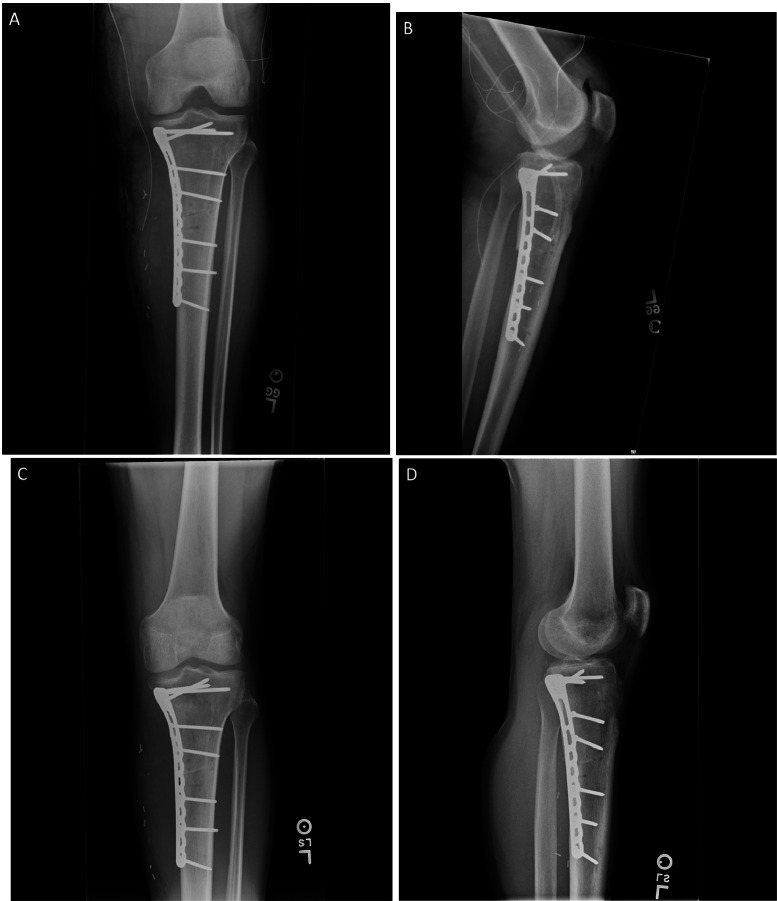
Postoperative radiographs of the left tibia status postsurgical resection of proximal tibial mass with allograft reconstruction. (A and B) Anteroposterior and lateral radiographs obtained 4 days after surgery show a nearly perfect fit of the allograft with medial plate and allograft fixation screws in place. (C and D) Anteroposterior and lateral radiographs obtained 5 months after surgery show progressive incorporation of the allograft.

The decision was made to proceed with wide surgical resection with allograft reconstruction given the histologic diagnosis, size, appearance, and rapid growth of the mass. Based on preoperative histologic diagnosis there was no indication for any systemic neoadjuvant treatments. Prior to surgery, the CT scan and the MRI of the surgical area were used in combination to plan the resection angles and to develop custom 3D printed cutting jigs (Onkos Surgical, Parsippany-Troy Hills, New Jersey) (Fig. 7). Additionally, these advanced images were used to obtain an allograft that would have the best fit for the patient's own anatomy (Fig. 8). After the cutting jigs were developed and the allograft identified, the patient underwent wide margin surgical resection through an open approach about the proximal medial tibia. The tumor was carefully dissected away from the soft tissues. Once there was adequate visualization, the custom cutting jigs were applied and their position was confirmed on imaging intraoperatively. The tumor was then resected and sent to pathology for further analysis. The custom allograft was then cut to fit the defect created by the resection and secured with plate and screws (Figs. 9A and B). The incision was closed primarily and the patient was placed in a brace locked in 30 degrees of flexion to allow for the medial collateral ligament to heal. The patient was admitted and made non weight bearing to allow for healing of the allograft initially. Over the course of 5 months postoperatively the patient regained their preoperative knee range of motion and was able to ambulate without pain. Postoperative radiographs obtained over their postoperative course demonstrated progressive healing of the allograft and native bone junction sites (Figs. 10A-D).

**Fig. 11:**
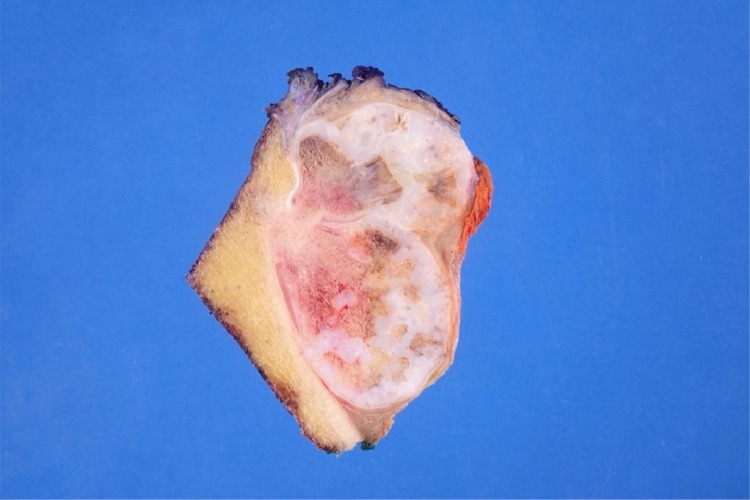
Gross photograph showing the cut surface of the resection specimen. An exophytic lobulated mass with a thick irregular cartilaginous cap arises from the bone surface without medullary involvement.

**Fig. 12:**
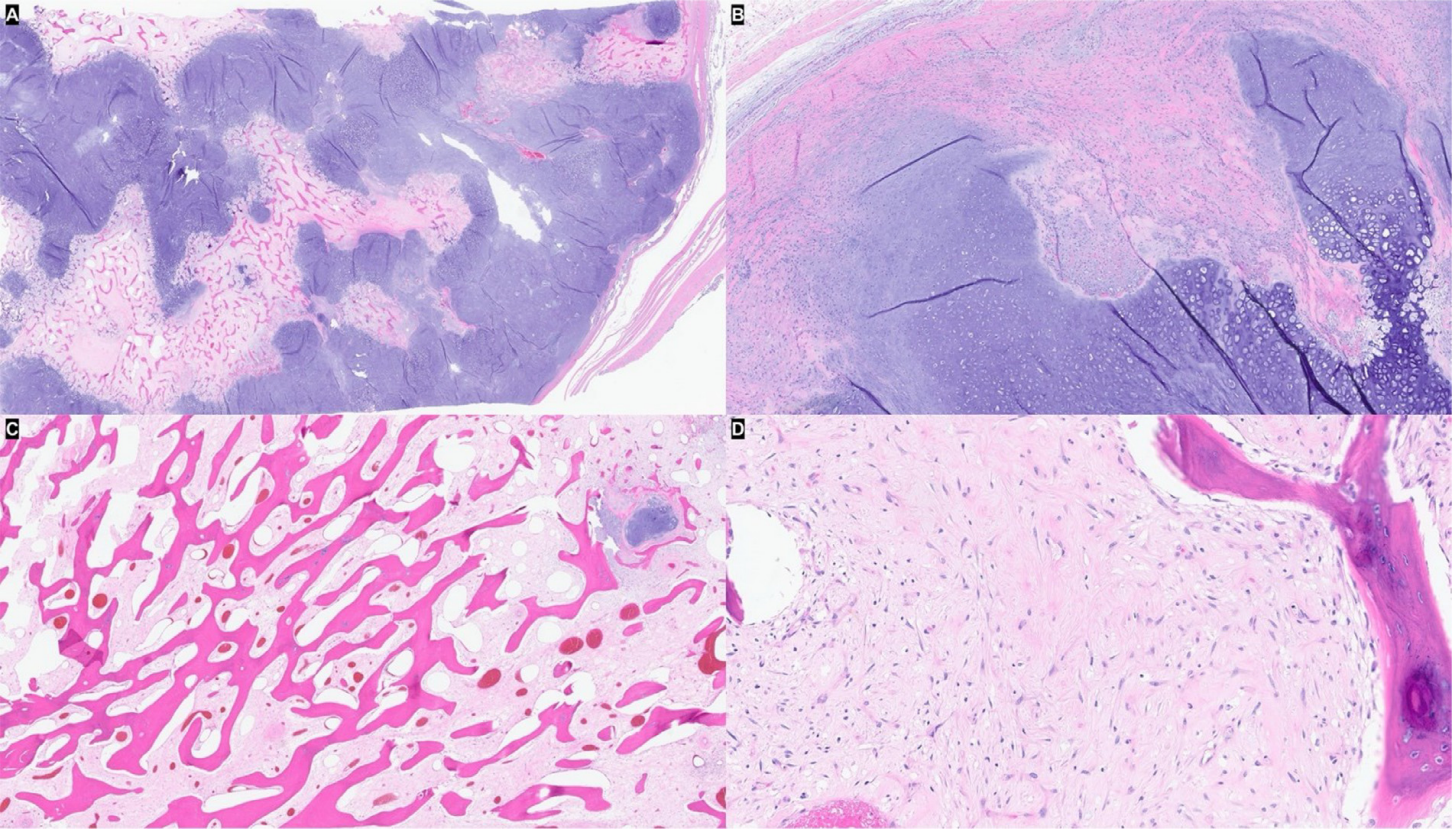
Photomicrographs of the resection specimen. (A) Thick irregular hyaline cartilage cap with anastomosing bone trabeculae (H&E, 16x). (B) The surface of the cap is irregular and ill-defined with hypercellular perichondrial fibrous tissue (H&E, 100x). (C) The osseous stalk is composed of linear anastomosing bone trabeculae with intertrabecular fibrous tissue (H&E, 40x). (D) Paucicellular intertrabecular fibrous tissue with atypical spindle cells (H&E, 400x).

Gross evaluation of the surgical specimen showed an exophytic lobulated mass arising from the tibial surface with an irregular cartilaginous cap (Fig. 11). Histopathologic evaluation demonstrated a prominent cartilaginous component mimicking the cartilage cap of an osteochondroma. However, unlike an osteochondroma, wherein the intertrabecular tissue of the stalk consists of adipose tissue, the intertrabecular tissue of the stalk in this tumor consisted of paucicellular fibrous tissue with atypical spindle cells and occasional mitoses (Figs. 12A-E). The underlying cortex was intact, another finding which argued against an osteochondroma. Based on these features, a final diagnosis of cartilaginous-rich parosteal osteosarcoma was established. This entity is also known in the literature as osteochondroma-like PO.

## Discussion

Osteochondroma-like PO is a rare variant of PO with only a handful of cases reported in the literature. In 1998, Lin et al. first described this entity when they presented 6 cases, all initially misdiagnosed as benign lesions—4 as osteochondromas, 1 as Trevor disease, and 1 as myositis ossificans [14]. All 6 of their initial biopsies showed a distinct cartilage cap with underlying mature bone, and it was discovered that the malignant components of these tumors were located in the periphery of the mass. The 6 cases were treated with surgical excision, and 4 of them developed recurrent disease eventually. Three other reported cases of osteochondroma-like PO from 2 separate case reports demonstrated the presence of cartilage at the periphery and emphasized the importance of evaluating the base of these tumors histologically, as sampling of the periphery can lead to misdiagnosis [3,4]. All three cases were initially misdiagnosed as osteochondromas. This highlights the variable distribution of the malignant features and cartilage components in osteochondroma-like PO, making it a challenge to diagnose with needle biopsy alone. Of the three cases, two were treated with surgical excision and one was treated with surgical excision and adjuvant radiation therapy. None had disease recurrence. Unique variants of PO can further complicate diagnosis. Cases of PO with an unexpected fatty component resembling atypical lipomatous tumor, both low grade chondrosarcoma and liposarcoma components, focal fatty metaplasia, and arising from beneath the periosteum have all been documented [2,[15], [16], [17]]. Such potential heterogeneity in composition and appearance necessitates a high index of suspicion and a thorough examination beyond initial biopsy findings when suspected PO is not confirmed histologically. Of note, these 4 cases were also treated with surgical excision without subsequent disease recurrence.

Our case is the first reported case in the literature of an osteochondroma-like PO that was initially diagnosed as low-grade periosteal chondrosarcoma. The initial biopsy sample was composed of mainly bland hyaline cartilage and was discordant with imaging findings. Needle biopsies are inherently limited in that they preclude assessing architectural features which can only be appreciated in an intact resection specimen. These features include the surface interface appearance, arrangement of linear anastomosing trabeculae, and relationship to the underlying cortex and medullary cavity. In addition, the cytologically atypical spindle cells may not be included in a needle biopsy sample. Since PO does not usually have a significant cartilage component, our initial needle biopsy was misleading.

One consideration to potentially improve the biopsy yield of suspected PO is to perform the biopsy with contrast-enhanced CT guidance, which can identify enhancing solid components of the tumor and guide biopsy of these areas. MDM2 and CDK4 immunohistochemical stains may provide additional support for the diagnosis of PO. However, these stains can be difficult to interpret and may not be helpful if the histologic appearance is not suggestive of PO, as with the initial biopsy of this case. Fluorescence in situ hybridization (FISH) testing for MDM2 amplification can also be valuable but, similar to immunohistochemistry, sampling of the lesional atypical spindle cells is necessary. As these cells were not present in our biopsy sample, ancillary testing likely would not have altered the initial diagnosis. Additional tissue sampling may have revealed atypical spindle cells to suggest PO and allow for FISH testing. However, given the prominent cartilaginous component of this tumor, we do not believe we could have obtained a definitive diagnosis of PO without surgical resection.

The surgical management in our case was innovative, utilizing imaging and software to custom-print 3D cutting guides tailored to the patient's anatomy. The utilization of custom cutting guides facilitated precise resection of the tumor and subsequent reconstruction of the tibia with an allograft, which was also customized to the patient, showcasing the potential of 3D printing technology in improving surgical outcomes and precision. The use of 3D printing in orthopedic surgery has already demonstrated promising results. Compared to conventional surgical methods, use of 3D printing has been shown to improve operative and fluoroscopy times, blood loss, bone union time, pain, accuracy, and function, without an increased complication rate [18]. Despite these advantages, many limitations still do exist including material availability, implant size and shape, cost, long production times, regulatory and safety issues, and limited evidence on implant longevity [[19], [20], [21]].

In conclusion, our case of an osteochondroma-like PO highlights the deceptive nature of these tumors on needle biopsy and reinforces the notion that definitive diagnosis may require surgical resection. Being aware of this entity, along with other variants of PO and the diagnostic pitfalls they present, is vital in navigating the complexities inherent in diagnosing these tumors. Multidisciplinary discussions and strong radiology-pathology correlation is essential for accurate diagnosis. Moreover, evolving surgical techniques that incorporate advanced technologies such as 3D printing for preoperative planning and reconstruction are enhancing patient outcomes, providing a beacon of progress in the surgical management of complex bone tumors.

## Patient consent

Written informed consent for publication of this case was obtained from the patient.
